# Monitoring of system conditioning after blank injections in untargeted UPLC-MS metabolomic analysis

**DOI:** 10.1038/s41598-019-46371-w

**Published:** 2019-07-08

**Authors:** Teresa Martínez-Sena, Giovanna Luongo, Daniel Sanjuan-Herráez, José V. Castell, Máximo Vento, Guillermo Quintás, Julia Kuligowski

**Affiliations:** 1 Hepatología Experimental, Health Research Institute La Fe, Valencia, Spain; 2 Health and Biomedicine, Leitat Technological Center, Valencia, Spain; 3 Centro de Investigación Biomédica en Red de Enfermedades Hepáticas y Digestivas (CIBERehd), Instituto de Salud Carlos III, Madrid, Spain; 4 Departamento de Bioquímica y Biología Molecular, Universidad de Valencia, Valencia, Spain; 5 Unidad Analítica, Health Research Institute La Fe, Valencia, Spain; 6 Neonatal Research Unit, Health Research Institute La Fe, Valencia, Spain; 7 Division of Neonatology, University & Polytechnic Hospital La Fe, Valencia, Spain

**Keywords:** Data acquisition, Metabolomics

## Abstract

Ultra-performance liquid chromatography – mass spectrometry (UPLC-MS) is widely used for untargeted metabolomics in biomedical research. To optimize the quality and precision of UPLC-MS metabolomic analysis, evaluation of blank samples for the elimination of background features is required. Although blanks are usually run either at the beginning or at the end of a sequence of samples, a systematic analysis of their effect of the instrument performance has not been properly documented. Using the analysis of two common bio-fluids (plasma and urine), we describe how the injection of blank samples within a sequence of samples may affect both the chromatographic and MS detection performance depending on several factors, including the sample matrix and the physicochemical properties of the metabolites of interest. The analysis of blanks and post-blank conditioning samples using t-tests, PCA and guided-PCA provides useful information for the elimination of background UPLC-MS features, the identification of column carry over and the selection of the number of samples required to achieve a stable performance.

## Introduction

Ultra-performance liquid chromatography coupled to mass spectrometry (UPLC-MS) is one of the most powerful and widely used techniques for conducting untargeted metabolomic analysis in biomedical research^1,2^. Recent developments in chromatographic columns such as narrow bore columns packed with sub-2 µm particles and instruments operating at pressures up to ~1400 bar have enhanced the chromatographic resolution and efficiency enabling better separation in less analysis time. Additionally, the development of high resolution MS instruments such as time of flight (TOF) or Orbitrap detectors, has expanded applications of highly sensitive and selective untargeted metabolomics to almost every area of biochemical research.

Raw UPLC-MS data must be appropriately handled to generate consistent, reproducible and meaningful data, with the reproducibility of metabolomic data requiring the implementation of correct quality assurance and quality control (QC) procedures, typically by the injection QC samples and blanks^3–10^. The repeated analysis of biological samples by UPLC-MS leads to an unavoidable interaction of matrix components and metabolites with different elements of the analytical system. This interaction leads to changes in the instrumental response such as retention time (RT) drifts, a decrease in the ionization performance and a decrease in sensitivity due to buildup of dirt on ion optics in the mass spectrometer. Because of this, after routine maintenance of the analytical system or the replacement of the analytical column, UPLC-MS metabolomic experiments demand an initial pre-conditioning of the equipment to reach a steady performance state, which is usually achieved by repeated injections of reference pooled samples^10–12^. The number of injections required to achieve this steady state of the analytical system largerly depends on the matrix and analytical procedure and is difficult to estimate in advance.

A second important issue in analytical pre-processing is the elimination of background interference signals arising from e.g. plasticizers, solvent impurities or reagents used during sample clean-up, as well as cumulative carryover contamination^10^. The elimination of these uninformative features improves the data quality as well as the relevance and the interpretability of generated models. This step also reduces the data set dimensionality, which in turn reduces the likelihood of observing chance correlations that limit the reliability of the results. But alongside these benefits, unavoidably some challenges arise related to deconditioning of the UPLC-MS system, which in turn can result in systematic variations in the metabolomic profiles of samples injected after the blank. Hence, the relative position of blanks within a sample batch must be established to minimize the impact on overall data quality. Broadhurst *et al*.^10^ have recenty recommended that the timing of blank injections should be correlated with initial column conditioning in order to measure systematic contamination, and with the end of the analytical batch to measure cumulative carryover. The authors also recommend to determine the number of QC samples required to condition the system in each laboratory as it is dependent on a number of factors including injection volume, blank solvent composition, sample type and instrument. Despite its importance, there is no standard way of assessing system conditioning, which is not trivial, as different approaches may lead to different conclusions. In this sense, there is a lack of agreement in the literature and the number of QCs injected recommended for system reconditioning vary considerably^10,13–16^.

In this article, using the analysis of two common bio-fluids (plasma and urine) and a set of straightforward univariate and multivariate methods for the assessment of system reconditioning, we describe how the injection of blank samples within a sequence of samples may affect both, the chromatographic and MS detection performance depending on several factors, including the sample matrix and the physicochemical properties of the metabolites of interest. This result highlights the importance of monitoring and ensuring a complete system reconditioning after each blank injection. Besides, results show that the analysis of blanks and post-blank reconditioning samples provides information useful to optimize background and carry over elimination and support the design of subsequent measurements with the aim of minimizing signal variability.

## Experimental Section

### Standards and reagents

Acetonitrile (CH_3_CN, LC-MS grade) was obtained from Scharlau (Barcelona, Spain) and formic acid (HCOOH, ≥95%) from Sigma-Aldrich Química SL (Madrid, Spain). Ultra-pure water was generated by a Milli-Q Integral Water Purification System from Merck Millipore (Darmstadt, Germany). Phenylalanine-D_5_, tryptophan-D_5_ and caffeine-D_9_ were purchased from C/D/N Isotopes Inc. (Quebec, Canada).

### Sample preparation

Ethical approval for this study was obtained from the Ethics Committee for Biomedical Research of the Instituto de Investigación Sanitaria Hospital Universitario y Politécnico La Fe (Valencia, Spain) (approval code EudraCT: 2014-000537-24) and all methods were performed in accordance with the relevant guidelines and regulations. A blood and a urine sample were collected from a volunteer who had given written informed consent to participate in the study.

3 mL of heparinized blood was centrifuged at 1500 × *g* (10 min, 4 °C). The plasma layer was collected and further centrifuged at 2500 × *g* (15 min, 4 °C). 600 μL of the plasma fraction were withdrawn and 1800 μL of cold (4 °C) CH_3_CN was added for protein precipitation. The sample was homogenized (Vortex shaker, 10 s) and centrifuged at 10000 × *g* (10 min, 4 °C). 1000 μL of the supernatant was collected and evaporated under vacuum at 25 °C. The residue was reconstituted in 700 μL of a 1 μM injection standard solution containing phenylalanine-D_5_, tryptophan-D_5_ and caffeine-D_9_ in H_2_O:CH_3_CN (98:2, 0.1% v/v HCOOH).

The urine sample was centrifuged at 2500 × *g* (10 min, 4 °C) and 250 μL of the supernatant were diluted with 500 μL of an injection standard solution containing phenylalanine-D_5_, tryptophan-D_5_ and caffeine-D_9_ in H_2_O:CH_3_CN (98:2, 0.1% v/v HCOOH) at a final concentration of 1 μM. Extraction blanks were prepared replacing blood or urine by MilliQ water and applying the same standard operating procedure as for plasma or urine samples.

### UPLC-ESI(+)-TOF-MS analysis

Chromatographic analysis was performed using a reversed phase Kinetex C_18_ (50 × 2.1 mm, 1.7 µm, Phenomenex) column on an Agilent 1290 Infinity UPLC chromatograph equipped with an iFunnel quadrupole time of flight (QTOF) Agilent 6550 spectrometer (Agilent Technologies, CA, USA). Autosampler and column temperatures were set to 4 °C and 55 °C, respectively, and the injection volume was 4 µl. Binary gradient elution (channel A: H_2_O, 0.1% v/v HCOOH, channel B: CH_3_CN, 0.1% v/v HCOOH) was performed at a flow rate of 400 µl min^−1^ as follows: initial conditions of 98% A followed by a linear gradient from 2% to 20% B in 0.3 min and from 20% to 95% B in 2.2 min. 95% B was held for 3.75 min and then, a 0.15 min gradient was used to return to the initial conditions, which were held for additional 1.6 min. Standard needle wash was carried out between injections using H_2_O:CH_3_CN (1:1 v/v) as solvent to reduce autosampler carryover from one sample to the next. Full scan MS data from 100 to 1700 *m/z* was acquired using the following positive electrospray ionization parameters: gas T, 200 °C; drying gas, 14 L min^−1^; nebulizer, 37 psig; sheath gas T, 350 °C; sheath gas flow, 11 L min^−1^. Automatic MS spectra calibration during the analysis using the 149.02332 (*phthalic anhydride*), 121.050873 (purine) and 922.009798 (HP-0921) *m/z* as references was carried out introducing a reference standard into the source *via* a reference sprayer valve. Data obtained during initial column conditioning was not included from data analysis.

An aliquot of the plasma and urine pooled samples was repeatedly analyzed using the auto MS/MS method with the following inclusion *m/z* precursor ranges: 70–200, 200–350, 350–500, 500–650, 650–800, 800–950, 950–1100 and 1100–1200 using, in all replicates, centroid mode at a rate of 3 spectra/s in the extended dynamic range mode (2 GHz). Collision energy was set to 30 V, MS/MS fragmentation with automated selection of three precursor ions per cycle and an exclusion window of 0.25 min after two consecutive selections of the same precursor. This way, a total of 2661 and 2776 MSMS spectra from the analysis of the plasma and urine QC were collected to support putative metabolite identification by matching the acquired spectra and the HMDB and LipidBlast reference databases using in-house software and LipiDex, for the automatic identification process^17^.

### Batch design

**Figure 1:**
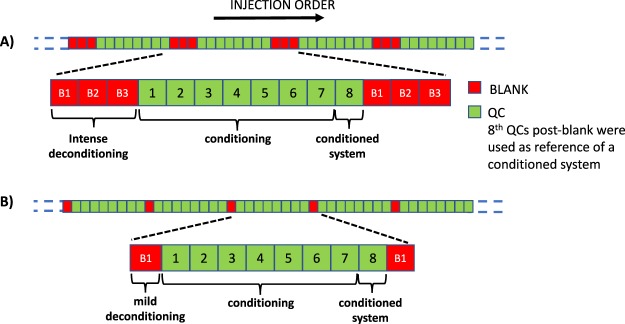
Scheme of the experimental design employed, in which either three consecutive (**A**) or one single blank sample (**B**) are analyzed after every 8 sample replicates for an intense or mild system deconditioning, respectively.

The objective of the batch design was the monitoring of the conditioning process after a blank injection in order to assess its effect on the system performance. For each matrix, two types of repeated cycles of system deconditioning/conditioning in a single batch were carried out. The first type used three consecutive blank injections for an intense deconditioning followed by 8 repeated sample injections for conditioning (see Fig. 1A). The second type used a single blank injection leading to a mild deconditioning also followed by 8 sample injections for system conditioning (see Fig. 1B). Plasma and urine analysis were carried out in two independent batches. A set of 10 QC replicates was injected at the beginning of each batch to ensure system conditioning. Between batches, the ESI interface was cleaned and the instrument was calibrated according to manufacturer recommendations. The urine set included data from the analysis of 156 urine samples and 37 blanks. The plasma data set included 136 replicate injections of a plasma sample and 36 blanks. The lower number of samples included in the plasma batch was due to an ‘instrument communication error’that caused the worklist to fail, stopping sample acquisition.

### Peak table generation and within-batch effect correction

Peak table generation was carried out for each batch (i.e. urine and plasma analysis) separately using XCMS software^18^. The *centWave* method was used for peak detection with the following parameters: mass accuracy: 20 ppm, peak width: (5,25), snthresh: 12, prefilter: (5,5000). A minimum difference in *m/z* of 7.5 mDa was selected for overlapping peaks. Intensity weighted *m/z* values of each feature were calculated using the *wMean* function. Peak limits used for integration were found through descent on the Mexican hat filtered data. Grouping before and after RT correction was carried out using the *nearest* method and 9 s as *rtCheck* argument. Finally, missing data points were filled by reintegrating the raw data files in the regions of the missing peaks using the *fillPeaks* method. Peak area, RT and peak widths, calculated as the difference between the end and start of the integration points, were extracted from XCMS data for each sample and UPLC-MS feature.

The accuracy of the peak integration and alignment was assessed by comparing peak area values obtained by manual and automated (i.e. XCMS) integration results for deuterated injection standards and known metabolites, obtaining linear correlation coefficients >0.998. The precision, as %RSD, associated with the injection standards across the entire injection sequences in raw data was: Phe-D_5_ 4.8% and 3.7% in samples and blanks (plasma batch), and 10.7% and 13.4% in samples and blanks (urine batch); Trp-D_5_ 3.4% and 4.3% in samples and blanks (plasma batch), and 7.2% and 11.7% in samples and blanks (urine batch), and Caff-D_9_ 2.6% and 6.4% in samples and blanks (plasma batch), and 13.8% and 11.5% in samples and blanks (urine batch).

Samples injected before a blank were used as reference (i.e. QCs) for the correction of within-batch effects to facilitate the interpretation and comparison of changes in the instrumental responses caused by system deconditioning. Within batch effect correction was carried out using the non-parametric Quality Control – Support Vector Regression Correction (QC-SVRC) approach employing a Radial Basis Function kernel, as described elsewhere^19,20^. QC-SVRC requires the selection of three structural hyperparameters: the tolerance threshold (ε), the penalty term applied to margin slack values (C) and the kernel width (*γ*). The selection was carried out using a pre-selection of C and optimization of *ε* and *γ* using a grid search, leave-one-out cross validation and the RMSECV as target function. C was selected for each UPLC-MS feature as the median value of the intensities observed in QC replicates. The *ε* search range was selected based on the expected instrumental precision (3–10% of the median value of the intensities observed for the whole set of QC replicates). The *γ* search interval selected was [1, 10^5^].

Data clean-up was carried out after within-batch effect correction using the set of blank samples for each batch independently. UPLC-MS features were classified as ‘informative’ and retained for further analysis if the following conditions were fulfilled: (i) the ratio between the minimum values in biological samples and the maximum value in blanks is >9; (ii) the peak area in more than 90% of the complete set of biological replicates was larger than the 9 times the maximum value in blanks, and iii) RSD(QCs) < 20% after batch effect correction. The first, second and third conditions identified 905, 950, and 3294 informative features in the plasma data set, and 2031, 2042, and 3387 in the urine data set. Data cleaning left 858 and 1991 features labelled as ‘informative’ in the plasma and urine data sets, respectively, for further data analysis.

### Guided PCA

Reese *et al*.^21^ proposed a δ statistic based on PCA and guided PCA for the statistical evaluation of batch effects in multivariate data sets that has demonstrated its applicability in high throughput genomics^21^ and metabolomics^22^. The δ statistic is defined as the ratio of the variance of the first principal component calculated using guided-PCA to the variance of the first principal component calculated from PCA as described elsewhere^21^. High values of δ (i.e. close to 1) are indicative of a batch effect and its statistical significance can be estimated by a permutation test in which the distribution of samples between batches are randomly permuted *m* times (here, *m* = 1000). For each permutation, a δ statistic is calculated (δ_p_). The δ value calculated using the real batch ordering is compared to the reference distribution of δ_p_ values and a one-sided *p*-value is estimated as:

$$\delta (p \mbox{-} value)=\frac{{\sum }_{m=1}^{M}(\hat{\delta } < \widehat{{\delta }_{p})}}{m}$$

In this work, we adapted the batch concept of guided-PCA, to evaluate the evolution of the system reconditioning through the use of replicate injections. A series of conditioning sets corresponding to the set of samples injected after *n* conditioning replicates were defined. Then, each conditioning set of replicates was compared paired-wise using the aforementioned guided-PCA approach to the set of samples injected after 8 conditioning replicates as reference (e.g. the set of sample replicates injected in 1^st^ position after a blank *vs* the set of samples injected in 8^th^ position after a blank).

### Software

Peak detection, integration, deconvolution and alignment were carried out using XCMS software^18^ in R 3.4.1. Principal component analysis (PCA), guided PCA, hierarchical cluster analysis (HCA) and univariate (t-test, False Discovery Rate (FDR) adjusted *p*-values using the Benjamini-Hochberg procedure^23^) analysis were carried out in MATLAB 2017b (Mathworks Inc., Natick, MA, USA) using in-house written scripts and the PLS Toolbox 8.3 (Eigenvector Research Inc., Wenatchee, USA). Support Vector Regression models were carried out in MATLAB using the LIBSVM library^24^. The datasets and scripts generated and/or analyzed during the current study are available from the corresponding author on reasonable request. Raw UPLC-MS data in mzXML format are also accessible via the MetaboLights repository (www.ebi.ac.uk/metabolights) under accession number MTBLS906.

## Results and Discussion

### Plasma data set

**Figure 2:**
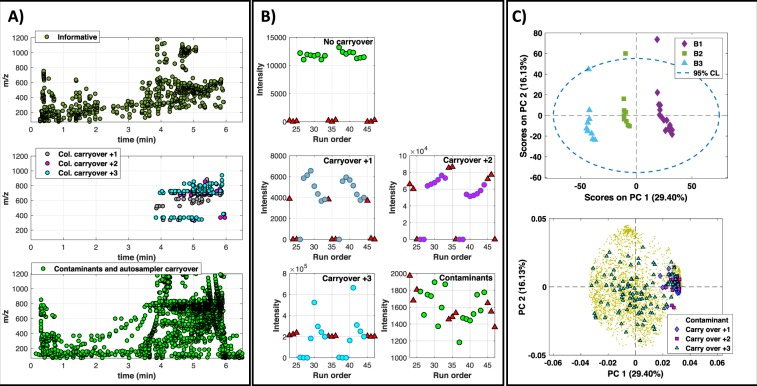
(**A**) Analysis of the distribution of informative, and uninformative (background contaminant and carryover) features in the plasma data set. (**B**) Representative intensity profiles in blanks and post-blank samples of UPLC-MS features labelled as informative, column carry over and contaminants; (**C**) PC1 *vs* PC2 scores (top) and loadings (bottom) from the PCA of UPLC-MS profiles obtained from the analysis of blanks, using features labelled as carryover or contaminant.

Carry over effects and background contamination are two sources of error that should be assessed and prevented during method development. Carry over is caused by analytes from a previously analyzed sample that were adsorbed on the autosampler or on the UPLC column. Autosampler carry over has a similar RT to that of the analyte and introduces a typically low but positive bias in subsequent samples that can be identified in blanks. Column carry over leads to features with unknown RTs that may stabilize after several injections and often generates random errors that affect the method precision^25^. Initial data pre-processing identified 2843 UPLC-MS features (76% of the total) for which the ratio between the minimum values in biological samples and the maximum value in blanks is <9. This set of uninformative features was analyzed to identify whether they arise from contamination and autosampler carry over, or column carry over. Column carry over was identified by assessing the relative position of missing values in plasma samples and blanks. Features that were detected in the 2^nd^ to 8^th^ sample and in the 1^st^ blank but neither in the first post-blanks sample nor in the 2^nd^ and 3^rd^ blank, were classified as ‘carry over +1’. The same approach was used to label features as ‘carry over +2’ (i.e. only detected in the 3^rd^–8^th^ post-blank samples and first and second blanks) and ‘carry over +3’ (i.e. only detected in the 4^th^–8^th^ post-blank samples and all blanks). Features detected in both, plasmas and blanks without following the abovementioned sequential patterns were classified as contaminants including e.g. plasticizers, and autosampler carry over. Figure 2A shows the distributions of the informative, column carry over and contaminant UPLC-MS features in the plasma data set. Features classified as ‘column carry over’ showed RTs in the 3.8 to 6 min range and included 44, 30 and 110 features classified as ‘carry over +1’, ‘+2’ and ‘+3’, respectively. Contaminants and informative features were distributed throughout the chromatogram. Figure 2B shows representative peak area profiles of informative, column carry over and contaminant features within two cycles of sample-blank injections.

Then, a PCA of data acquired from blanks excluding the informative features, was carried out. The generated overview of the data structure was then used to identify trends and clusters in the data set and to find which samples and UPLC-MS features were most strongly correlated with each PC. PCA scores plot depicted in Fig. 2C showed a close grouping of blank injections according to their relative run order along PC1. Loadings plot included in Fig. 2C identified column carry over ‘+1’ and ‘+2’ features as highly correlated to blanks injected immediately after a sequence of samples (i.e. Blank #1 in the figure).

**Figure 3:**
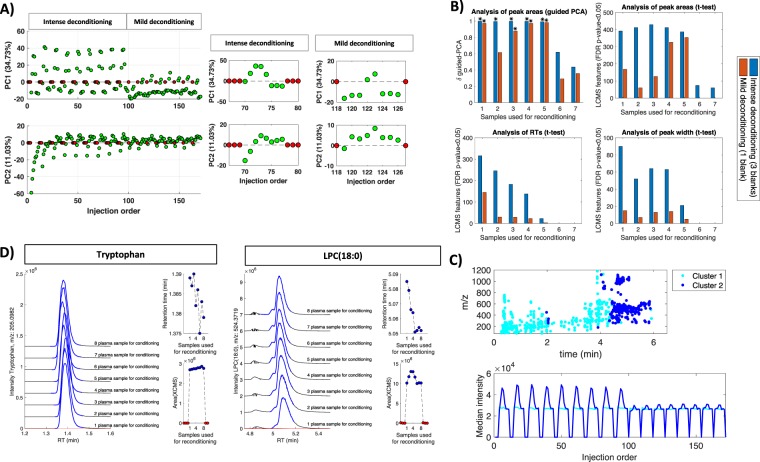
Analysis of the effect of blank injections during the analysis of a batch of plasma samples. (**A**) PCA scores (PC1 and PC2) as a function of the injection order. Red circles indicate the position of the blanks; (**B**) Evolution of the δ value and the number of UPLC-MS features showing different mean peak area, RT and peak width using as reference the mean values calculated after the injection of 8 samples for reconditioning. Note: *indicates permutation test p-value < 0.05; (**C**) Distribution (top) and mean intensity profiles (bottom) of the features included in clusters 1 and 2 (see text for details); (**D**) Peak area values, RT and extracted ion chromatograms for tryptophan (included in HCA cluster #1) and LysoPC(18:0) (included in HCA cluster #2) in plasma replicates. Red dots: blanks. Colored bold lines in the chromatograms indicate the limits of the XCMS integration window.

The exploratory analysis of the data set excluding blanks as well as uninformative features (i.e. labelled as contaminants or carryover) was carried out by PCA. Figure 3A shows PC1 and PC2 scores from a PC model accounting for 45.7% of the total variance of peak area values. In the case of blanks having no effect on the system’s performance, the plot of the PCA scores should not display any association with the injection order in the sample replicates following a blank injection. However, results depicted in Fig. 3A showed repeatable trends. After an intense system deconditioning by injecting three blanks consecutively (e.g. injections #70–77, see Fig. 3A), PC1 scores increased and reached a maximum value after the injection of 3–4 biological samples before returning to the initial value after 6–7 biological samples. PC2 scores decreased after the injection of blanks and then rapidly increase to reach the original value after the injection of 6–7 biological samples. The effect of mild system deconditioning after a single blank injection was less intense and returned to stable PC1 and PC2 values after 2 plasma injections, PC1 scores increase after 4–5 plasma injections and then returned to the initial value.

Visual inspection of PCA scores plots is widely used to generate an overview of the structure of multivariate data sets. However, PCA is an unsupervised method and the effect of system deconditioning after a blank injection could remain undetected if they are not among the largest sources of data variability. Results depicted in Fig. 3B indicate that, in this case, results using the δ statistic calculated using guided PCA were in agreement with those obtained by visual inspection of PC scores during intense deconditioning. A significant difference (δ > 0.8, *p*-value < 0.005) was found between the reference set of samples injected in 8^th^ and those in the 1^st^–5^th^ positions post-blank. Results showed a successful reconditioning after the injection of 6 samples (δ < 0.6, *p*-value > 0.05).

Thereafter, a HCA of the row-wise normalized UPLC-MS peak area profiles as a function of the injection order was carried out for the identification of characteristic deconditioning-conditioning profiles using the Pearson correlation coefficient as distance measure for clustering (see Fig. 1S, left). The distributions of *m/z* and RT values of the UPLC-MS features included in the two main clusters were markedly different (see Fig. 3C), indicating that the impact of the system deconditioning was not homogeneous across the chromatogram. While cluster #1 included 452 UPLC-MS features (52.7% of the total) with RT in the 0.5–4.5 min range, cluster #2 included 406 UPLC-MS features covering mainly lipophilic compounds of high molecular weight eluting at RT > 4.5 min. Median normalized intensity profiles of the two main clusters depicted in Fig. 3C were in agreement with the stabilization of the response after 6–7 reconditioning samples showed by multivariate (PCA) and univariate (t-test) analysis (see Fig. 3A,B): on the one hand, mean peak area profile of UPLC-MS features in cluster #1 showed a minor increase in peak area values after the blank and a recovery of the initial intensity after 4–5 sample injections. On the other hand, the mean peak area profile of UPLC-MS features included in cluster #2 showed a gradual increase in intensity after a blank injection, reaching a maximum intensity 3–4 injections after the blank, recovering the initial intensity after 6–7 reconditioning injections. These intensity profiles matched with patterns observed for features assigned to column carry over (see Fig. 2B), indicating that co-elution with column carry over features may be an additional factor affecting the UPLC-MS profiles.

**Figure 4:**
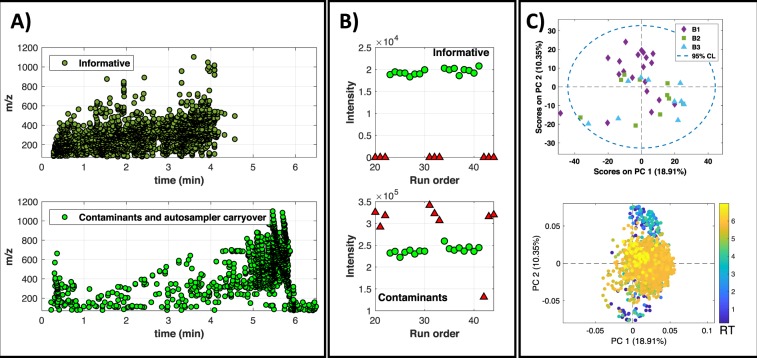
(**A**) Analysis of the distribution of informative, and uninformative (background contaminant and carryover) features in the urine data set. (**B**) Representative intensity profiles in blanks and post-blank samples of UPLC-MS features labelled as informative or contaminants; (**C**) PC1 *vs* PC2 scores (top) and loadings (bottom) from the PCA of UPLC-MS profiles obtained from the analysis of blanks, using features labelled as carryover or contaminant.

Changes in the intensity, RT and peak widths of the UPLC-MS variables after a blank were also assessed by a univariate approach using the set of plasma samples analyzed in 8^th^ position after a blank as reference of a conditioned system. The evolution of the number of UPLC-MS features showing different peak area distributions (t-test, unequal variances, FDR *p*-value < 0.05) as a function of the number of plasma samples used to recondition the system is shown in Fig. 3B. Results showed that the intensity of 392 UPLC-MS features (46% of the total) and 168 (19% of the total) after an intense or mild deconditioning, respectively - were affected by a previous blank injection. Although the number of UPLC-MS features affected by the system deconditioning was higher in case of an intense deconditioning, results indicate that 6–7 plasma replicates were required in both cases to reach stable analytical performance. Changes in XCMS corrected RT and peak width were also used as descriptors of the stability of the chromatographic performance after a blank injection. Results from t-tests depicted in Fig. 3B showed a post-blank change in the chromatographic performance that was more intense after an intense deconditioning, as shown by the higher number of features showing a shift in the RT and/or peak width. Although alignment during peak table generation adjusted for RT differences between runs, any change in RT might lead to differences in the ionization performance due to ion suppression or enhancement effects from partially co-eluting analytes. Figure 3D depicts the variation in the extracted ion chromatograms, peak area and RTs observed for two metabolites included in cluster #1 (tryptophan, *m/z*-RT: 205.0982-1.38) and cluster #2 (LysoPC(18:0), *m/z*-RT: 524.3719-5.06)). Additional examples (LysoPC(18:2) (*m/z*-RT: 520.3422-4.66) and LysoPC(16:0) (*m/z*-RT: 496.3435-4.75)) can be found in Figs 3S and 4S. Data depicted in Fig. 3D shows that the blank injection increased the RT of LysoPC(18:0) from 5.05 to 5.09 min and modified the UPLC-MS response as shown by the change in relative intensity and resolution of the peak shoulder at 5.03 min. The RT shift correlates with the increase in the peak area values depicted in Fig. 3D, indicating a change in the system response for LysoPC(18:0) that is corrected after reconditioning using 6–7 plasma samples. These results were in agreement with the variation of the number of UPLC-MS features with significantly different peak widths and RTs shown in Fig. 3B, in this case, the chromatographic and MS performances were recovered after the injection of 5–6 samples. Notably, only a small variance was observed in terms of peak shape, RT or area value for tryptophan. Assessment of system reconditioning by guided PCA was carried out independently for each subset of UPLC-MS features included in both clusters. Results found showed again two different profiles (see Fig. S2). For variables included in cluster #1, statistically significant δ values (*p*-value < 0.05) were obtained when less 3 samples were used for reconditioning after the injection of ≥1 blank. The analysis for variables included in cluster #2 indicated that at least 7 sample replicates were required for a system re-equilibration after a mild deconditioning or intense deconditioning, respectively.

### Urine data set

Initial data pre-processing in the urine data set for the identification of informative, contaminant and carryover features was carried out as described before. Figure 4A shows the distributions of the UPLC-MS features labelled as informative (n = 2031) or contaminant (n = 1557) features in the urine data set. No feature was labelled as ‘column carry over’. Figure 4B shows representative peak area profiles of informative, column carry over and contaminant features within a cycle of sample-blank injections. Contaminants and informative features were distributed throughout the chromatogram.

**Figure 5:**
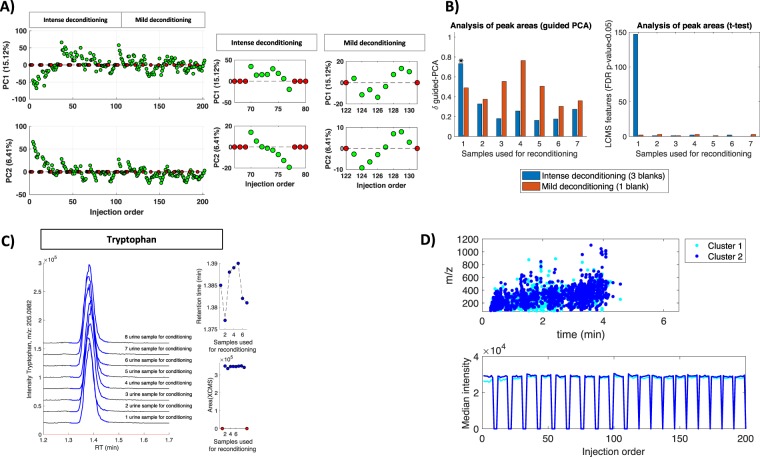
Analysis of the effect of blank injections during the analysis of a batch of urine samples. (**A**) PCA scores (PC1 and PC2) as a function of the injection order. Red circles indicate the position of the blanks; (**B**) Evolution of the δ value and the number of LC-MS features showing different mean peak area using as reference the mean values calculated after the injection of 8 samples for reconditioning. Note: *indicates permutation test p-value < 0.05; (**C**) Peak area values, RT and extracted ion chromatograms for tryptophan in urine replicates. Red dots: blanks. Colored bold lines in the chromatograms indicate the limits of the XCMS integration window; (**D**) Distribution (top) and mean intensity profiles (bottom) of the features included in clusters 1 and 2 (see text for details).

As the blanks and the urine are alike in polarity, the effect of an intense or mild deconditioning is minor compared to plasma. PCA scores plot depicted in Fig. 4C showed an incomplete grouping of blanks injected immediately after a sequence of samples and a high overlap between blanks injected in 2^nd^ and 3^rd^ position after a sequence of samples. Despite the lower separation in the PC scores space between blanks injected in 1^st^, 2^nd^ and 3^rd^ position, loadings plot identified a cluster of UPLC-MS features with RTs < 2 min as correlated to blanks injected in 1^st^ position after a sequence of samples. Results depicted in Fig. 5 showed the effects of the blank on the stability of the system, following the same strategy used for the analysis of plasma batch. In this case, visual inspection of PCA scores plot depicted in Fig. 5A did not show a consistent pattern of variation after the injection of one or three blanks the first two PC components.

Results using the δ statistic calculated using guided PCA only showed a statistically significant difference (δ *p*-value < 0.05) between the reference set of samples injected in 8^th^ and 1^st^ position after the injection of three consecutive blanks (see Fig. 5B).

Figure 5B also depicts results from *t*-test carried out using the set of samples analyzed after 8 sample replicates as reference indicating that, under these experimental conditions, the injection of a single blank within the sequence, did not have a significant effect on the performance of the analytical system. A more intense deconditioning modified the peak area values of up to 147 features (7.4%) (FDR t-test *p*-value < 0.05) and this number was reduced down to 1 features after injecting 2 or more samples for reconditioning.

Univariate analysis of changes in the XCMS corrected RT and peak width also showed a smaller effect than that observed during the analysis of plasma samples and no feature showed a significant change in the XCMS corrected RT or peak width (FDR t-test *p*-value < 0.05) after intense or mild deconditioning. Figure 5C shows as an example, the stability of the elution profile, RT and peak area of tryptophan after the injection of three blanks. Finally, multivariate clustering of the mean peak area profiles observed in urine samples lead to the identification of two main clusters (see Fig. S1, right). The plot of the *m/z* and RT distribution of the features included in both clusters depicted in Fig. 5D did not show any characteristic pattern which could be attributed to e.g. physico-chemical properties of the metabolites, in agreement with the depicted stable median intensity profiles.

## Conclusions

Correct conditioning of the analytical system and the elimination of background and carry over UPLC-MS features are important steps during method development and in the data pre-processing pipeline. In this article, we show that the analysis of blanks and conditioning samples using uni- and multivariate tools such as t-test, PCA and guided-PCA provides useful information for the elimination of background and carry over UPLC-MS features and for the selection of the number of samples required to achieve a stable performance.

Results show that reconditioning of the system after blank injection depends on several factors, including the extent of the deconditioning, the sample matrix and the physicochemical properties of the metabolites of interest. Patterns observed in plasma but not in urine samples during post-blank conditioning could be partially attributed to the stabilization of column carry over due an incomplete elution of the late eluting, lipophilic fraction of high molecular weight compounds. Sample preparation is a key aspect that influences the role the blank injections play in deconditioning the column. Although not investigated in this work, a more effective sample clean-up (e.g. improved protein precipitation) or the use of different chromatographic conditions (e.g. longer column re-equilibration, change in the mobile phase pH or flow rate) could influence the results found in this study including the number of QC samples required for conditioning.

The results of our work indicated that, with the analysis of system conditioning after the injection of blanks facilitates the select the number of QCs required for system reconditioning (e.g. 7 and 4 for plasma and urine samples, under the analytical conditions employed in this work) and supports the identification of carry over.

## Supplementary information

Supplementary info.
