# Supplementary material for: Monitoring of system conditioning after blank injections in untargeted UPLC-MS metabolomic analysis
Source: Sci Rep. 2019 Jul 8;9:9822. doi: 10.1038/s41598-019-46371-w (PMC6614502; doi:10.1038/s41598-019-46371-w)
Supplement: Supplementary file 1 — Supplementary info. [file 41598_2019_46371_MOESM1_ESM.pdf]

# Monitoring of system conditioning after blank injections in untargeted UPLC-MS metabolomic analysis

Teresa Martínez-Sena[a], Giovanna Luongo[a], Daniel Sanjuan-Herráez[b], José V. Castell[a,c,d,e], Máximo Vento[f,g], Guillermo Quintás[b,e*], Julia Kuligowski[f]

[a]*Hepatología Experimental, Health Research Institute La Fe, Valencia, Spain*

[b]*Health and Biomedicine, Leitat Technological Center, Valencia, Spain*

[c]*Centro de Investigación Biomédica en Red de Enfermedades Hepáticas y Digestivas (CIBERehd), Instituto de Salud Carlos III, Madrid, Spain*

[d]*Departamento de Bioquímica y Biología Molecular, Universidad de Valencia, Valencia, Spain*

[e]*Unidad Analítica, Health Research Institute La Fe, Valencia, Spain*

[f]*Neonatal Research Unit, Health Research Institute La Fe, Valencia, Spain*

[g]*Division of Neonatology, University & Polytechnic Hospital La Fe, Valencia, Spain*

*e-mail: gquintas@leitat.org

**SUPPLEMENTARY MATERIAL**

**Figure S1.** HCA of the intensity profiles in the plasma (left) and urine (right) data sets using the Ward's algorithm for clustering and the Pearson coefficient as distance measure.

**Figure S2.** Assessment of system reconditioning using guided PCA in plasma. The analysis was carried out independently for each subset of UPLC-MS features included in clusters 1 (left) and 2 (right). Note: * indicates permutation test *p*-value<0.05.

**Figure S-3**. Peak area values, RT and extracted ion chromatograms for LysoPC(18:2) (included in HCA cluster #2) in plasma replicates. Red dots: blanks. Bold lines in the chromatograms indicate the limits of the XCMS integration window.

**Figure S-4**. Peak area values, RT and extracted ion chromatograms for LysoPC(16:0) (included in HCA cluster #2) in plasma replicates. Red dots: blanks. Colored bold lines in the chromatograms indicate the limits of the XCMS integration window.

Figure SM1.

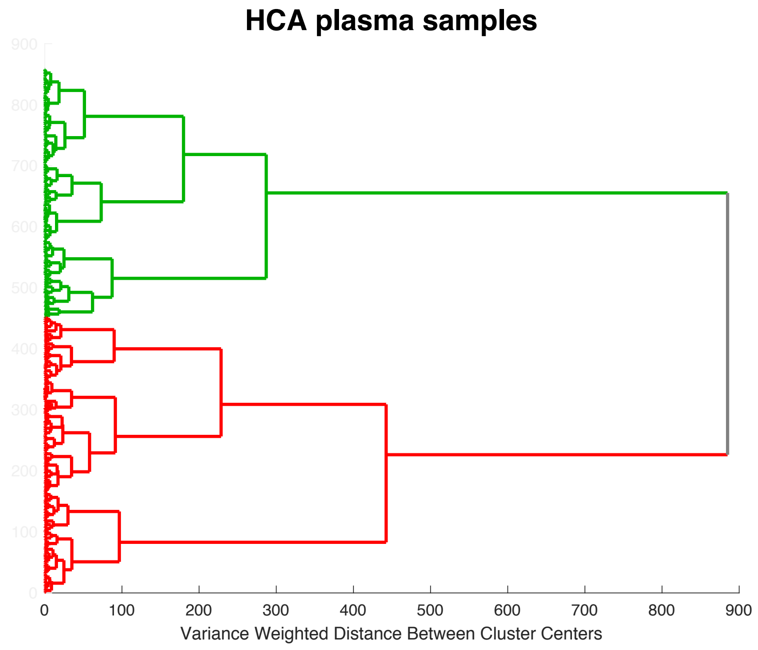
HCA plasma samples
900
800
700
600
500
400
300
200
100
0
0
100
200
300
400
500
600
700
800
900
Variance Weighted Distance Between Cluster Centers

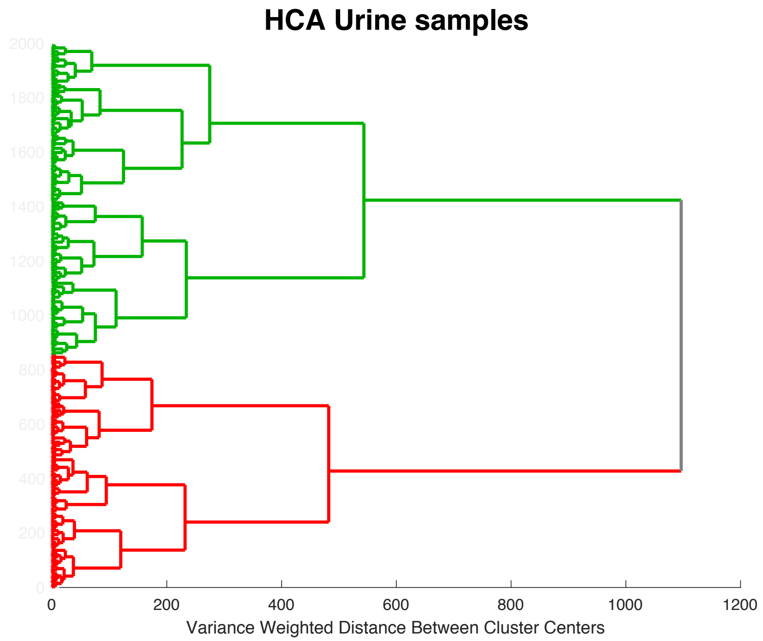
HCA Urine samples
2000
1800
1600
1400
1200
1000
800
600
400
200
0
0
200
400
600
800
1000
1200
Variance Weighted Distance Between Cluster Centers

Figure SM2.

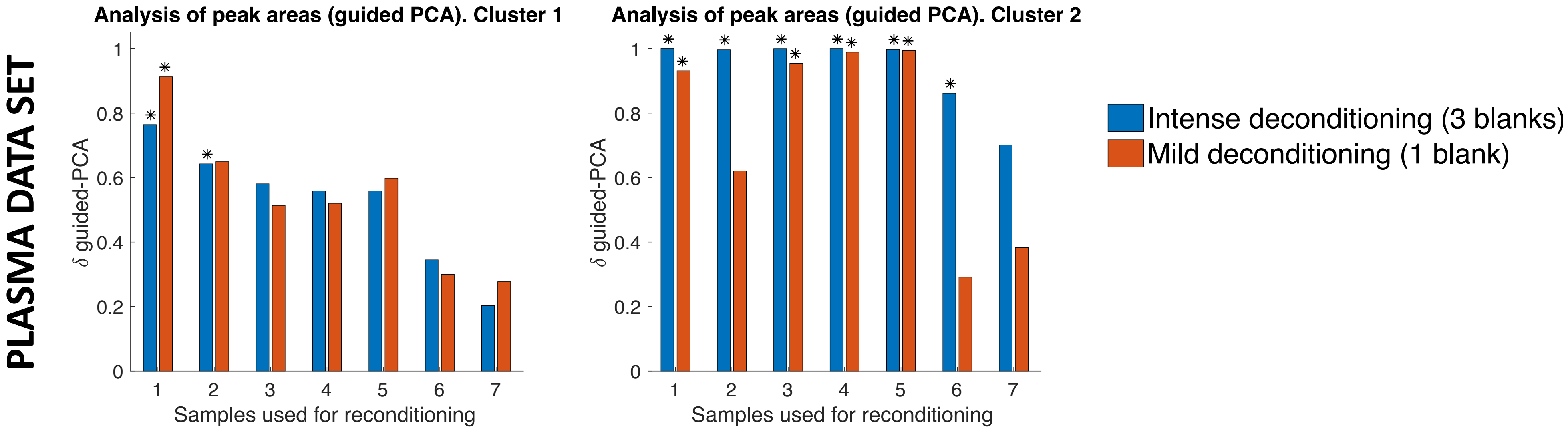
PLASMA DATA SET
Analysis of peak areas (guided PCA). Cluster 1
Analysis of peak areas (guided PCA). Cluster 2
δ guided-PCA
Samples used for reconditioning
Intense deconditioning (3 blanks)
Mild deconditioning (1 blank)

Figure SM3.

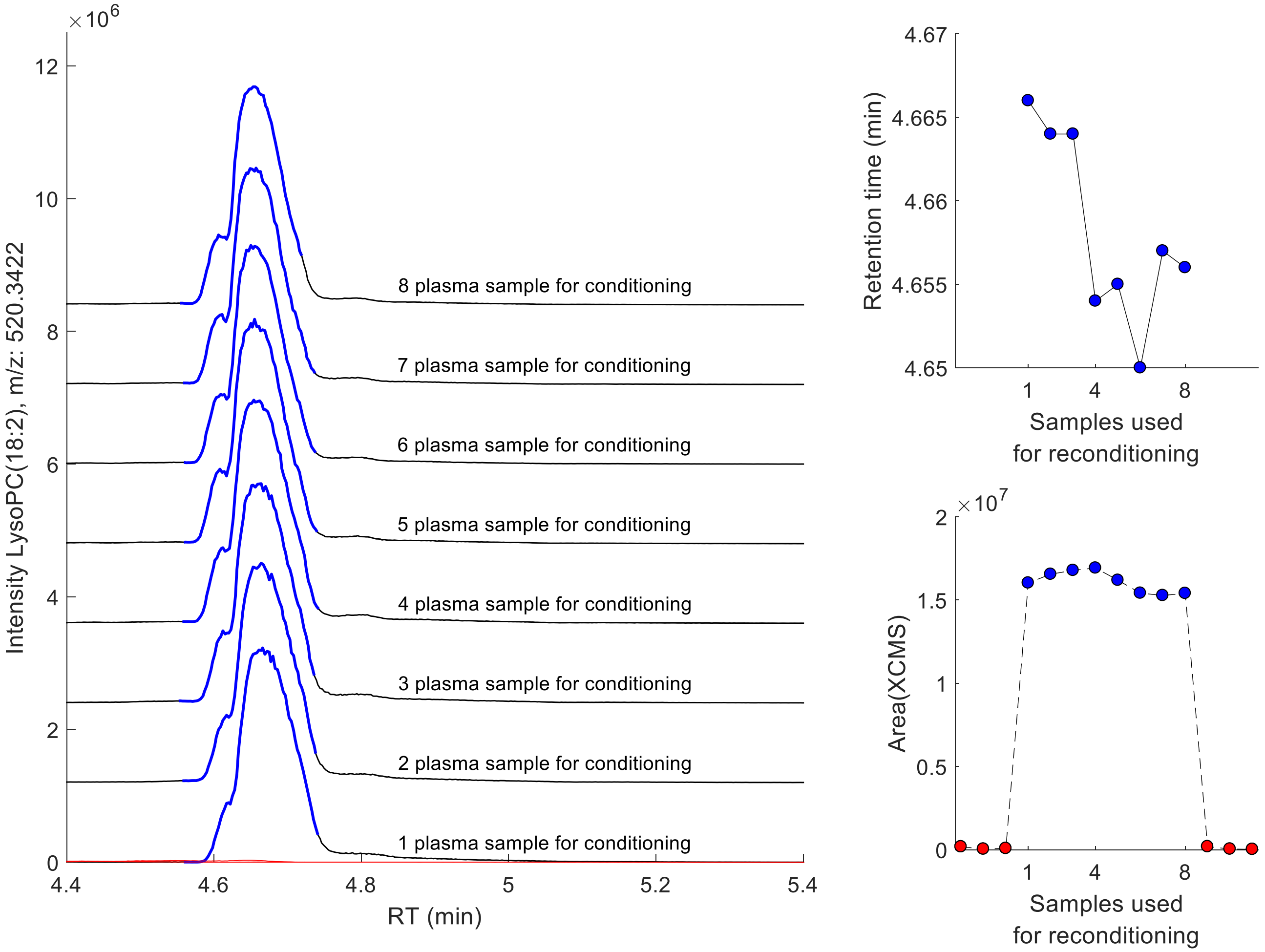

×10^6
12
10
8
6
4
2
0
Intensity LysoPC(18:2), m/z: 520.3422
8 plasma sample for conditioning
7 plasma sample for conditioning
6 plasma sample for conditioning
5 plasma sample for conditioning
4 plasma sample for conditioning
3 plasma sample for conditioning
2 plasma sample for conditioning
1 plasma sample for conditioning
4.4
4.6
4.8
5
5.2
5.4
RT (min)
4.67
4.665
4.66
4.655
4.65
Retention time (min)
1
4
8
Samples used
for reconditioning
×10^7
2
1.5
1
0.5
0
Area(XCMS)
1
4
8
Samples used
for reconditioning

Figure SM4.

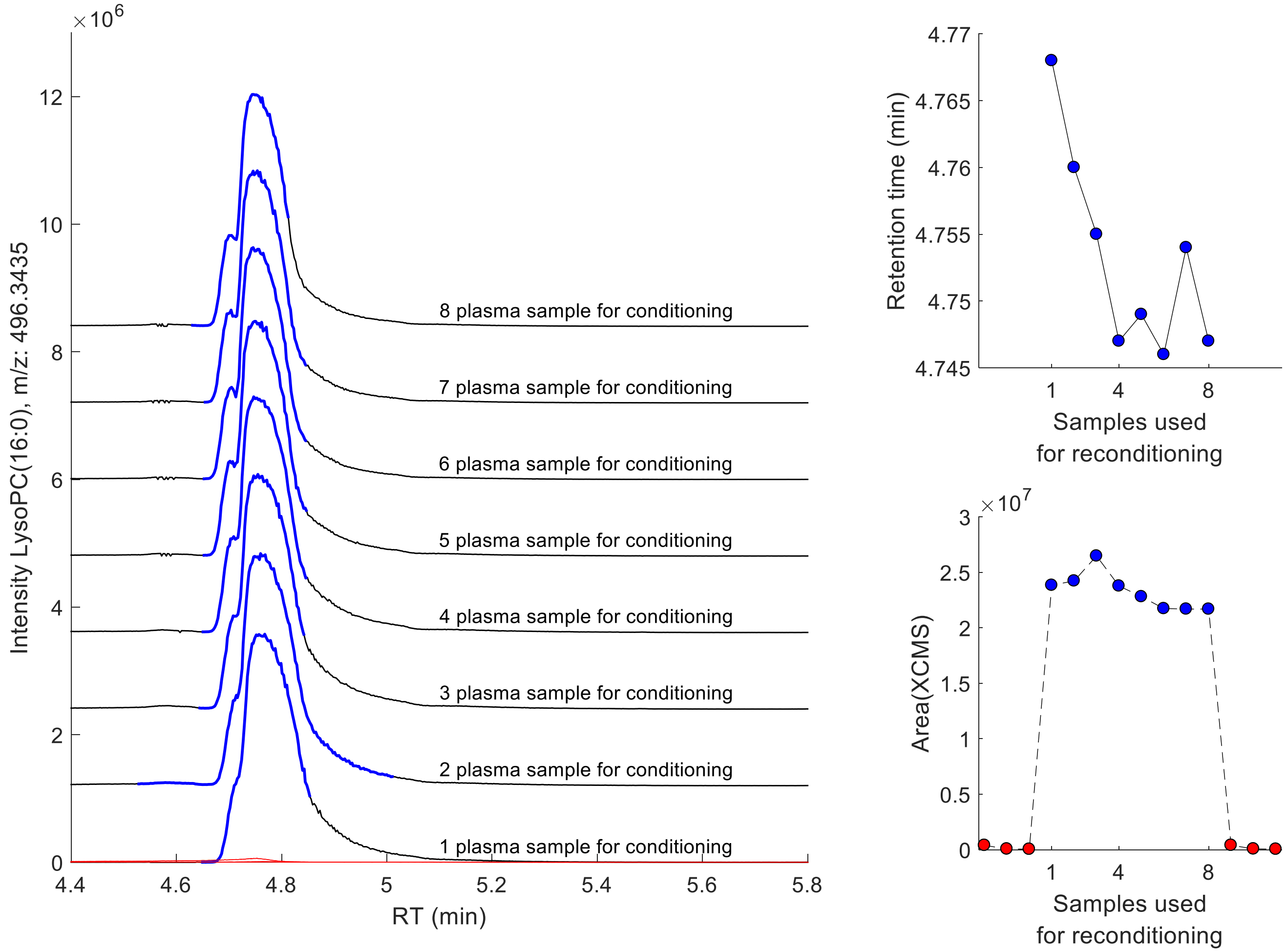

×10^6
12
10
8
6
4
2
0
Intensity LysoPC(16:0), m/z: 496.3435
8 plasma sample for conditioning
7 plasma sample for conditioning
6 plasma sample for conditioning
5 plasma sample for conditioning
4 plasma sample for conditioning
3 plasma sample for conditioning
2 plasma sample for conditioning
1 plasma sample for conditioning
4.4
4.6
4.8
5
5.2
5.4
5.6
5.8
RT (min)
4.77
4.765
4.76
4.755
4.75
4.745
Retention time (min)
1
4
8
Samples used
for reconditioning
×10^7
3
2.5
2
1.5
1
0.5
0
Area(XCMS)
1
4
8
Samples used
for reconditioning
